# Gingerols and shogaols: A multi-faceted review of their extraction, formulation, and analysis in drugs and biofluids to maximize their nutraceutical and pharmaceutical applications

**DOI:** 10.1016/j.fochx.2023.100947

**Published:** 2023-10-20

**Authors:** Yasmin R. Maghraby, Rola M. Labib, Mansour Sobeh, Mohamed A. Farag

**Affiliations:** a Department of Chemistry, The American University in Cairo, New Cairo, Egypt; b Pharmacognosy Department, Faculty of Pharmacy, Ain Shams University, Cairo, Egypt; c AgroBioSciences Program, Mohammed VI Polytechnic University, Lot 660, Hay Moulay Rachid, Ben-Guerir 43150, Morocco; d Pharmacognosy Department, College of Pharmacy, Cairo University, Cairo, Egypt

**Keywords:** Gingerols, Ginger, Extraction, Formulations, Antioxidant, Shogaols

## Abstract

Gingerols represent the main bioactive compounds in ginger drugs mostly *Zinigiber officinale* (F. Zingebraceae) and account for the biological activities and the strong/pungent flavor in ginger. Ginger (*Z. officinale*) rhizome is one of the most valued herbal drugs for ailments’ treatment in many ayurvedic medicine asides from its culinary applications as a spice. Gingerols and their dehydrated products shogaols are phenolic phytochemicals found in members of the *Zingiberaceae* family and account for most of their effects including anti-inflammatory and anticancer activities. This review entails most of the novel trends related to the extraction, optimization, and formulations of gingerols and shogaols to insure best recoveries and efficacies from their natural resources. Further, it presents a comprehensive overview of the different analytical approaches for the determination of gingerols/shogaols’ levels in nutraceuticals to ensure highest quality and for their detection in body fluids for proof of efficacy.

## Highlights

• Multifaceted review of gingerols and shogaols distribution in planta.
• Comparison of extraction methods to provide highest recovery of gingerols.
• Analytical methods used for detection in food and biofluids are described.
• Formulation of gingerols in nutraceuticals and food to improve its stability.
• This review provides data to improve health value and consumer acceptance of gingerols.

## 1 Introduction

**Fig 1:**
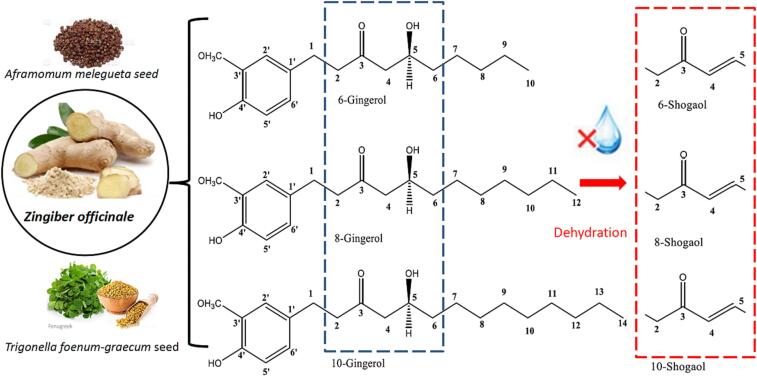
Structures of gingerols and their dehydrated derivatives shogaols.

Dietary sources present a rich source of phytochemicals that possess health-protective effects in addition to their nutritive value. Fruits, vegetables, herbs, and spices have grabbed the worldwide attention owing to their numerous health benefits (Li et al., 2019). In addition to macro- and phytonutrients, dietary sources encompass a broad range of biologically active phytochemicals most of which have been applied in traditional medicine for decades (Shukla & Singh, 2007). Among spices and herbal products, ginger (*Zingiber officinale* Rosc.) belongs to the tropical/sub-tropical family – *Zingiberaceae*’ that is becoming one of the most popular nutraceuticals worldwide aside from its culinary uses as spice. A typical ginger plant entails perennial, tuberous stems and rhizome that are acceptably applied as medications in Arabic, Indian and Asian ayurvedic medicine since long ago. Additionally, the plant was widely used in China for ca. 2500 years to treat common colds, nausea, headaches and is currently recognized in herbal medication practices especially for the treatment of muscular discomfort, rheumatological conditions, arthritis, etc. (Das et al., 2021). Several population-based studies revealed that people in Southeast Asia are at less risk of prostate, colon, breast, gastrointestinal cancers than their Western counterparts. It is believed that their diet’s constituents, including ginger, play a vital role in lowering cancer incidence (Mao et al., 2019). Ginger rhizome encompasses essential oil (i.e., 1 % to 3 %) being composed of terpenoids such as zingiberene and pungent phenolics such as, shogaols, zingerones, gingerols, etc., of which gingerols and shogaols are the key bioactives in ginger that account for most of its health effects. Other sources of gingerols *in planta*, thought at lower levels, include *Aframomum melegueta* (Zingiberaceae), seeds of *Trigonella foenum-graecum* L. (Fabaceae), and roots of *Lycianthes marlipoensis* (Solanaceae) (Li et al., 2019). Gingerols are typically a mixture of volatile phenolics, of which 6-gingerol is the main form, whereas 4-, 8-, 10- and 12-gingerols exist at lower levels. All gingerols’ classes are a series/chains of several homologs which are mainly distinguished by means of the unbranched alkyl chains’ length (Fig. 1) *viz.* 10-gingerol ((5S)-5-hydroxy-1-(4-hydroxy-3-methoxyphenyl) tetradecan-3-one), 8-gingerol ((5S)-5-hydroxy-1-(4-hydroxy-3-methoxyphenyl) dodecan-3-one) and 6-gingerol (5-hydroxy-l-[4-hydroxy-3-methoxyphenyl] decan-3-one) (Shukla and Singh, 2007, Li et al., 2019). All these compounds are thermally labile and are being extensively changed upon drying to shogaols by dehydration. This typically gives a pungent and spicy-sweet fragrance in dried ginger that lasts for years (Shukla & Singh, 2007). In contrast, thermal treatment of gingerols yields the pungent principle “zingerone”, also termed vanillylacetone as a main constituent of the ginger’s pungency, yet imparts the sweet flavor of both dry and cooked ginger (Das et al., 2021). Several reviews have addressed the biological effects and especially focused on the health benefits associated with gingerols (Dilukshi Vichakshana et al., 2022, Li et al., 2019, Promdam and Panichayupakaranant, 2022, Shukla and Singh, 2007). Suppl. Fig. S1 shows examples of ginger extract delivery methods, e.g., micro emulsification & oil-in-water macro emulsions, together with examples of their biological effects and uses. However, this review article gathers information about gingerols from multi perspectives. To elaborate, this review article presents a multifaceted updated overview of gingerols’ chemistry, extraction techniques, delivery systems, as well as analytical technologies for quality control purposes (Chari et al., 2013).

## 2 Gingerols/shogaols extraction and isolation

**Fig 2:**
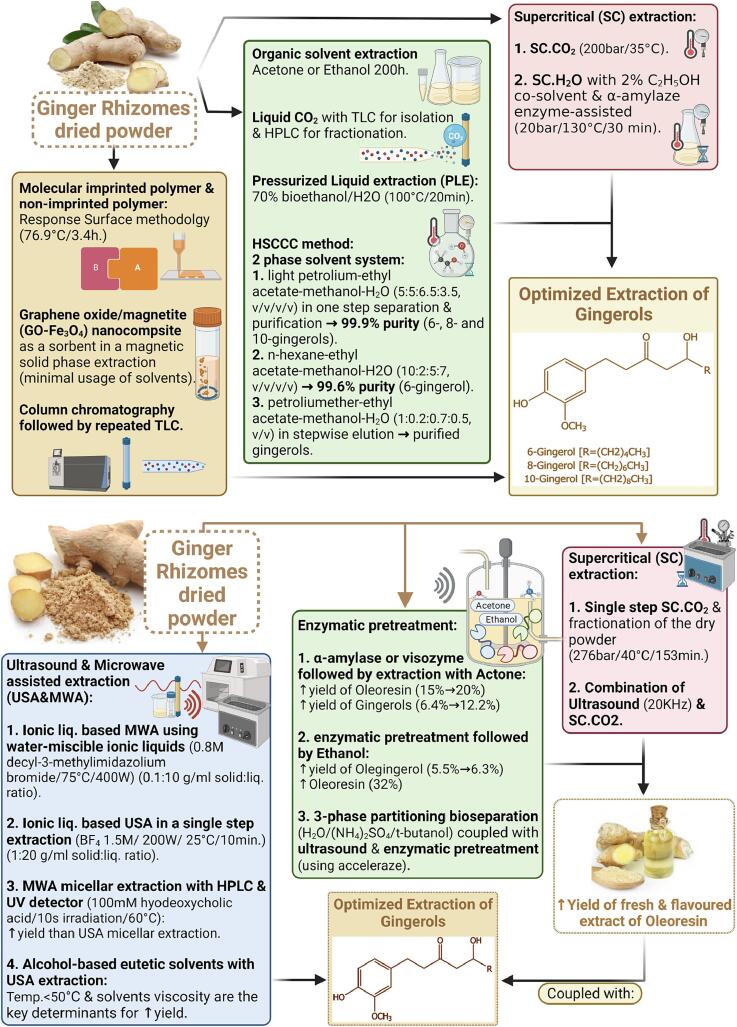
Schematic diagram of the main extraction methods used for ginger bioactives.

Several extraction methods were reported for gingerols either using organic solvents or more advanced extraction techniques, such as supercritical solvents, e.g., carbon dioxide alone or in combination with ultrasonic or microwave assisted extraction (Ginithillawala Arachchilage et al., 2022, Malik, 2019). This section highlights the application of each technique, advantages, and limitations if any with emphasis on the needed developments especially for upscaling to industrial levels as shown in Fig. 2.

### 2.1 Solvent extraction (SE)

Solvent extraction is a common method where chemical compounds are being separated based on their solubility using a solvent which has a special affinity towards the targeted molecules (Zou et al., 2022, Promdam and Panichayupakaranant, 2022). Conventional solvent extraction methods either using percolation or Soxhlet have led to improved gingerol yield, but unfortunately are rather time and solvent consuming (Shukla, Das, & Goud, 2020). Several reports examined the extraction of active ingredients from ginger by SE. For instance, a convectional SE method was applied to extract 6-, 8- and 10-gingerols and 6-shogaol from ginger, with best solvent for recovery of gingerols identified as 95 % ethanol having an extraction yield at 7.806 mg/g dried weight. Likewise, gingerols degradation products namely 6-shogaol was extracted using 95 % ethanol at different temperatures, i.e., room temperature, 60 °C or 80 °C, to identify the optimum extraction temperature. The highest yield of 6-shogaol was attained at 80 °C, with 7-folds higher levels of 6-shogaol compared to extraction at room temperature. The yield was reported at about 22 mg/g dry mass (Note et al., 2012). Another study tested the effectiveness in applying pressurized liquid extraction for ginger extraction using both water and bioethanol as solvents. Among different proportions of aqueous solutions of ethanol, 70 % ethanol showed optimum results in terms of recovery of gingerols and exaction yield where it yielded 12.2, 2.1 and 4.1 mg/g of 6-, 8- and 10-gingerol, respectively (Hu et al., 2011). A recent study optimized the extraction method of the bioactives from ginger utilizing response surface methodology that applied Box–Behnken design to a several independent variables including ethanol concentration (0–70 %), extraction temperature (50–70 °C extraction time (30–90 min). As a result of the study, the ethanol concentration (41.38 %), the extraction temperature (70 °C), and the extraction time (78.16 min) were considered the optimum conditions. Also, the class of ginger used also showed substantial effect on the amounts of recovered gingerols, yet its inclusive effect relied more on the type of solvent used (Liu et al., 2014). Keosaeng et al., compared several solvents for the extraction of 8- and 10-shogaol from ginger rhizomes, with hexane to provide the optimum recovery. 8-& 10-Shogaol were used as insecticidal agents against *Spodoptera* spp. larvae, with an LD50 of 9.92 and 8.40 µg/larva after 24 and 48 h, respectively. The extraction yields (wt./wt.) were found comparable for hexane, dichloromethane, ethyl acetate, and methanol ranging from 1.2 to 2 %, with hexane showing the best yield (Keosaeng et al., 2023).

### 2.2 Subcritical water extraction (SWE)

Compared to organic solvents that present hazardous effects towards the environment and towards us as well (Anisa et al., 2014), awareness in the application of more ecofriendly techniques is on the rise such as subcritical water extraction, which does not use organic solvents for the recovery of natural products (Ginithillawala Arachchilage et al., 2022). SWE is a novel and efficient method for extracting compounds with low polarity as in gingerols applying H_2_O solely for a rather short extraction duration time of *ca*. 30 min. The subcritical H_2_O is kept in the liquid phase with high pressure and at temperatures varying among 100 and 375 °C·H_2_O at elevated temperatures possess a low dielectric constant to weaken hydrogen bonds’ strength and renders subcritical H_2_O analogous to the polar organic solvents with less polarity, e.g., ethanol and methanol, while precluding any toxic solvents. Solubility of phenolic compounds of low polarity is improved at higher subcritical water’s temperature (Ko et al., 2020). Antioxidant capacity and percentile yield of SWE of soil grown versus soilless grown ginger were examined. The results showed that soil grown ginger exhibited 46 % more extract than the soilless grown plants. Soilless grown ginger yielded 440 g (1.46 %) of ginger extract versus 950 g (3.17 %) of ginger extract was yielded from the soil grown ginger. [6]-Shogaol was more dominant in soilless grown ginger, whereas [6]-gingerol content was more in soil grown ginger. Soil grown ginger showed greater antioxidant activities than soilless ginger (Razak et al., 2023). SWE was reported to extract bioactive compounds (i.e., gingerols and shogaol) from ginger. The effects of adding co-solvents, temperature, time as well as particle size on the extraction yields as well as the effect of enzyme and ultrasonic pretreatment were studied. By adding 2 % ethanol as a co-solvent, better extraction yield was attained, whereas optimal temperature/extraction time was attained at 130 °C and a duration of 30 min, respectively. Pretreatment with enzymes increased gingerols and shogaol yields detected at 5325 µg GAE/g dried ginger and 2990.5 µg/g dried ginger, respectively (Znourbakhsh Amiri et al., 2018). A recent report proposed a single step supercritical CO_2_ extraction-fractionation process for the production of volatile oil and gingerols-enriched oleoresin where the results showed that oleoresin was 96.15 wt% and was also enriched with 51.2 wt% of gingerols (Shukla et al., 2020a). Using supercritical fluid extraction CO_2_ at 15 MPa and 35 °C provided a 2.8 % yield from *Z. officinale* var. *Amarum* oil with 22.30 % 6-gingerol content (Salea, Veriansyah, & Tjandrawinata, 2017). Altogether, additional experiments are still needed to explore a wide range of different temperatures and pressures with respect to the thermally labile components (gingerols). The application of subcritical water as the extraction medium for gingerols isolation from ginger rhizomes was proven to be a superior technique providing higher yields. The research showed that the gingerol content of the extract was up to 5 % obtained using subcritical water extraction carried out at a temperature of 130 °C, a pressure of 3 bar, and an extraction time of 20 min (Myulianto et al., 2017).

### 2.3 Microwave-assisted extraction (MAE)

MAE is a typical procedure used for extracting phytochemicals from medicinal plants that relies on microwave energy to heat samples inside certain solvents, thus separating analytes from the sample matrices into the surrounding solvent. MAE main advantage lies in its capacity of rapidly heating the sample providing rapid extraction of analytes well suited for thermally unstable metabolites (Suktham et al., 2021). An ionic liquid-based MAE technique was applied to isolate gingerols and shogaols from ginger. Out of the tested ionic liquids, the results revealed that the carbon chains of alkyl cations significantly influenced the extraction efficiency of 6-gingerol and 1-decyl-3-methylimidazolium bromide was identified as an optimal ionic liquid. The study also revealed that upon comparing with an extraction yield of 0.595 % using methanol-based extraction and 0.673 % methanol-based MAE, ionic liquid-based MAE attained a higher yield of 0.716 % with an additional added value of a quicker extraction duration time of 30 min for the recovery of shogaols and gingerols from ginger (Guo et al., 2017). Liu et al. extracted 6-gingerol *via* MAE by comparing different microwave power (i.e., 200 W to 700 W). Improved extraction yield was observed with increase in the microwave power from 200 W and 500 W. Above a power of 500 W, the yield decreased suggestive that at high microwave loss of 6-gingerol occurs due to its degradation (Liu et al., 2014). In another study, MAE combined with an acidic food condiment was applied to augment levels of 6-, 8-, and 10-shogaols. Ginger was processed in an aqueous tartaric acid solution (0.8 mol/L, 8 mL) at a temperature of 140 °C and 1000 W for a duration of 10 min. The yields of 6-, 8-, and 10-shogaols (4.66, 1.19, and 1.76 mg/g dry mass, respectively) were detected at *ca*. 12-, 17-, and 19-folds more in processed ginger than in its unprocessed form as expected considering that shogaols represent the degradation products of gingerols. It was reported that processed form of ginger can be used as a good quality raw material for manufacturing different ginger commodities, besides for the preparatory purification and separation of 6-, 8-, and 10 shogaols (Guo et al., 2015). Additionally, MAE followed by UPLC/UV was applied for the extraction and identification of gingerols and shogaols from ginger. Zingerone, 6-gingerol, 8-gingerol, 6-shogaol and 10-gingerol were detected at 0.095, 2.368, 0.566, 0.905, 1.015 mg/g, respectively with 6-gingerol as the major component (Peng et al., 2017). In a further study, 6-gingerol and 6-shogaol were extracted using MAE with optimal conditions identified at ethanol concentration of 70 %, extraction time of 10 min, and microwave power of 180 W. Within the above-mentioned optimal parameters, the experimental values were 2.8 ± 0.6 mg/g 6-gingerol, 1.3 ± 0.5 mg/g 6-shogaol, respectively (Teng, Seuseu, Lee, & Chen, 2019).

### 2.4 Enzyme-assisted extraction (EAE)

Using enzymes in diverse industrial applications has long been acknowledged for their effectiveness, both economically and technologically *via* achieving high product yields and avoiding severe operational conditions. Additionally, enzymes are being increasingly reported in extracting phytochemicals as an environmentally friendly (i.e., green) extraction technique. EAE method employs certain classes of enzymes for the disruption of cell walls to ease the extraction and improve the yield (Das et al., 2021). The effects utilizing cellulase, protease, pectinase, α-amylase and viscozyme enzymes on the extraction yield of 6-gingerol were studied. It was observed that pre-treatment of ginger using α-amylase/viscozyme prior to extraction with acetone resulted in increased yield of gingerol (12.2 %) when compared with control (6.4 %). Extraction of ginger rhizome, that was pre-treated by enzyme followed by ethanol extraction resulted in elevated gingerols yields (6.2–6.3 %) compared with control (5.5 %). In addition, the ethanolic extract of cellulase pre-treated ginger showed optimum contents of polyphenols at 37.5 mg/g (Chari et al., 2013). Another study used cellulases for the extraction of gingerol oleoresin. Cellulase pretreatment led to a substantial increase in 6-gingerol and 6-shogaol yields in comparison with untreated control detected at 6152 and 2855 (ug.g^−1^) (Varakumar et al., 2017). Noteworthy, EAE is a potential method for the recovery of gingerol as it uses low temperature; however, additional experiments are required to explore the combination of two or more enzymes together as well as other extraction techniques, among them SWE.

### 2.5 Ultrasonic-assisted extraction (UAE)

UAE has been increasingly used as a speedy and efficient extraction method that employs ultrasound to provide fast movement of the solvent, creating a higher mass transfer speed and acceleration of the extraction process (Sharayei et al., 2021). An ultrasonic-assisted production of an alcohol based deep eutectic solvent was investigated for the extraction of gingerols from ginger powder. Markedly, certain prepared alcohol based deep eutectic solvent showed better extracting performances than using classical organic solvents. 6-,8- and 10-gingerols were detected at 0.014, 0.022 and 0.019 mg/g, respectively (Hsieh et al., 2020). UAE coupled with micellar extraction was applied in one of the studies resulting in the extraction of zingerone, 6-gingerol, 8-gingerol, 6-shogaol, and 10-gingerol. Surfactants were used to solubilize solutes in water and to facilitate their separation. The results showed that limits of detection were in the range of 3.8–8.1 ng/mL and that average recoveries ranged from 87.3 to 103.1 % (Peng et al., 2017b). In another study, ionic liquid-based UAE was employed for gingerols’ extraction. The parameters identified to mostly affect recovery of gingerols included solid/liquid ratio, extraction temperature, ionic liquid concentration, ultrasonic power, ionic liquid type, and extraction time. Compared to the traditional extraction techniques, ionic liquid-based UAE significantly increased total gingerols’ yield concurrent with less extraction time suggesting that ionic liquid-based UAE presents a potential technique for the extraction of gingerols in single step, with a yield of extraction of total gingerols reported at 12.2 mg/g (Kou et al., 2018). Extraction of 6-gingerol using sonic assisted water extraction at both low (28 kHz) and high frequencies (800 kHz) was examined. Applied power, followed by the entrainer, extraction time, sample to solvent ratio, frequency, temperature, and, lastly, mean particle size (MPS) were identified as the most significant parameters. MPS 0.89– 1.77 mm, 45 min, 40 W applied power, 1:30 (w/v), 45 °C and 10 % of ethanol as entrainer were also found to be the optimum conditions for high frequency SAWE prototype. At these conditions, high frequency SAWE yielded a recovery that was 2.7 folds higher in comparison to that obtained by low frequency SAWE. Thus, high frequency sonication for thermolabile compounds was found to be more effective than low frequency SAWE at optimized parameters. The effects of low and high frequencies on ginger cells were tested utilizing a SEM and effects of high frequency were milder compared to those of low frequency. The concentration of 6-gingerol obtained using high frequency SAWE was almost the same as other extraction methods except in case of accelerated solvent extraction (ASE), which was much higher. High frequency SAWE could be considered as a possible extraction method for thermolabile compounds with milder operating conditions. The potential of adding entrainer to sonication assisted extraction should be further explored as it was identified previuosly to enhance recovery significantly (Syed Jaapar, Morad, Iwai, & Nordin, 2017). Jan at al. applied UAE to extract polyphenols from freeze-dried ginger at an extraction efficiency of 15.3 %. However, it was observed that ethyl acetate/ethanol is needed to obtain extracts with better antioxidant capacity. Several types of phytoconstituents were found in extracts, e. g., alkaloids, tannins, phenols, flavonoids, etc. (Jan et al., 2022) which have yet to be optimized for individually.

### 2.6 Magnetic solid-phase extraction (MSPE)

MSPE is receiving increasing attention lately, with several advantages compared with the standard extraction approaches as the magnetic/magnetizable sorbent is disseminated in the sample’s solution thus increasing the interfacial area among the sample and the sorbent (Jiang et al., 2019). MSPE presents magnetic field that can lessen sample pretreatment procedure with no need for centrifugation and filtration. Generally, magnetic adsorbents are recycled easily presenting an added value in terms of being economic and ecofriendly as a green extraction method in addition to its robustness, speediness posing it for large scale industrial level (Jiang et al., 2019). In a study, graphene oxide magnetite nanoparticles were produced and applied as sorbents for the extraction of gingerols from commercial tea samples, ginger extracts, ginger candies, fresh ginger, tonic water, and thermogenic supplements. Recoveries from ginger extracts were reported at 4, 0.3, 0.3 ug mg^−1^ for 6-gingerol, 8-gingerol, and 10-gingerol, respectively. MSPE presented accuracy, environmental-friendly and efficient extraction techniques for determining gingerols (Akamine et al., 2021). Table 1 summarizes main features of each extraction methods applied for gingerols and shogaols.

**Table 1:** Different extraction methods reported for gingerols and shogaols.

Methodology	Bioactive compounds	Results	Ref.
Solvent extraction (SE)	Total gingerols	7.80 mg/g dry weight	(Liu et al., 2014)
	6-Shogaol	22 mg/g dry mass	(Note et al., 2012)
	6-, 8- & 10-Gingerol	12.2, 2.1 and 4.1 mg/g yield of 6-, 8- & 10-gingerol, respectively	(Hu et al., 2011)

Subcritical water extraction (SWE)	Total gingerols & shogaols	2990 & 5 µg/g dried ginger, respectively	(Znourbakhsh Amiri et al., 2018)
	Gingerol	5 % gingerol content in was obtained using SWE carried out at a temperature of 130C, pressure of 3 bar and extraction time of 20 min.	(Myulianto et al., 2017)

Supercritical fluid extraction CO_2_	6-Gingerol	Using 15 MPa and 35 °C, 2.8 % yield from *Z. officinale* var. *Amarum* oil with 22.3 % 6-gingerol content	(Salea et al., 2017)

Microwave-assisted extraction (MAE)	6-, 8-, 10-Gingerols & 6-, 8-, 10-shogaols	0.71 % extraction yield of shogaols & gingerols	(Guo et al., 2017)
	6-, 8- & 10-Shogaols	6-, 8- & 10-shogaols yields at 4.6, 1.1 & 1.7 mg/g dry mass	(Guo et al., 2015)
	Zingerone, 6-gingerol, 8-gingerol, 6-shogaol and 10-gingerol	Zingerone, 6-gingerol, 8-gingerol, 6-shogaol and 10-gingerol detected at 0.095, 2.4, 0.56, 0.9, 1.0 mg/g	(Peng et al., 2017)
	6-Gingerol and 6-shogaol	6-gingerol and 6-shogaol were extracted using MAE at an optimal conditions of 70 % ethanol, extraction time 10 min, and microwave power of 180 W. Within the above-mentioned optimal ranges, experimental values were 2.8 ± 0.6 mg/g 6-gingerol, 1.3 ± 0.5 mg/g 6-shogaol, respectively	(Teng et al., 2019)

Enzyme-assisted extraction (EAE)	6-Gingerol	12.2 % Extraction yield	(Chari et al., 2013)
	6-Gingerol & 6-shogaol	6-Gingerol & 6-shogaol were extracted at 6902 and 2752 (ugg^−1^)	(Varakumar et al., 2017)

Ultrasonic-assisted extraction (UAE)	6-, 8- & 10-Gingerol	6-gingerol, 8- & 10-gingerol detection limits were at 0.014, 0.022 & 0.019 mg/g	(Hsieh et al., 2020)
	Total gingerols	12.21 (mg/g, dried weight)	(Kou et al., 2018)
	6-Gingerol	Optimum conditions for high frequency SAWE prototype applied were MPS 0.89– 1.77 mm, 45 min, 40 W applied power, 1:30 (w/v), 45 °C and 10 % of ethanol as entrainer. Results yielde a concentration and recovery that was 2.69 times higher compared to results using low frequency SAWE.	(Syed Jaapar et al., 2017)

Magnetic solid-phase extraction	6-, 8- & 10-Gingerol	4 µg mg^−1^, 0.29 µg mg^−1^, 0.3 µg mg^−1^ for 6-, 8-, and 10-gingerol	(Akamine et al., 2021)

## 3 Quality control analysis of gingerols and shogaols

**Fig 3:**
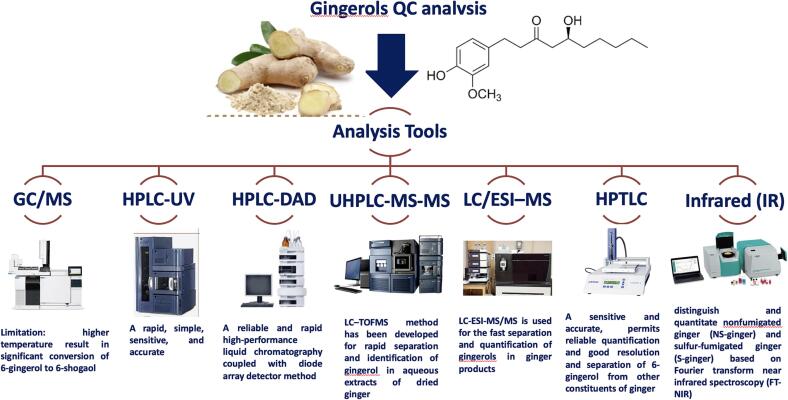
Chromatographic and spectroscopic techniques used for the detection of bioactives form ginger highlighting their main features.

Different chromatographic techniques have been reported in the literature for detection of gingerols and shogaols either qualitatively or quantitatively *viz.* HPTLC, GC/MS, HPLC/UV, LC/MS, IR, and electrochemical devices (Fig. 3 & Table 2). These approaches’ main goal is to provide a rapid, reliable, and sensitive method for their detection in natural sources as well as biofluids for proof of efficacy. The next subsections shall provide a brief of the different techniques highlighting their applications, advantages and or any limitations.

**Table 2:** Different analytical techniques employed for the analyses of gingerol and shogaols.

Technique	Sample	Goal	Results	Ref.
HPTLC	*Z. officinale* rhizome (shade-dried and powdered)	Asses for accuracy, precision of the method, and gingerol recovery	HPTLC method provided quantitative determination of 6-gingerol*.* The method is fast, simple, and precise, that can be used in regular quality testing	(Rai, Mukherjee, Mal, Wahile, Saha, & Mukherjee, 2006)
	Fresh ginger (3 varieties), splashed with water, further oven dried at (50⁰C) then powdered	Design a low cost, fast and simple densitometer procedure for the detection of 6-, 8-, 10-gingerol and 6-shogaol in ginger samples	The suggested densitometric procedure is satisfactory for the regular investigation of ginger samples in laboratories.	(Melianita et al., 2007, Melianita et al., 2009)
	Dried whole rhizome of *Z. officinale*, ginger root dietary supplement, two ginger rhizome teas and two ginger commercial creams.	Quantification of 8-gingerol	HPTLC detected gingerol at a concentration range of 50–500 ng/spot. Accuracy was at 98.2 % with RSD 0.70–1.4.	(Alam, 2013)
	*Z. officinale* fresh rhizomes from various locations in India	Develop and validate fast RP HPTLC. Separation of all 3 gingerol peaks	The ease of preparation method, robustness in a short period makes HPTLC superior technique for fingerprint analyses.	(Khan et al., 2010)
	Samples of ginger rhizome, commercial ginger powder, commercial capsules, and commercial ginger teas were extracted by conventional and ultrasonication-assisted methods	Determination of 6-shagaol and 6-gingerol	R_f_ = 0.36 for 6-shagaol 0.53 for 6-gingerol. The 6-shagaol content was determined in samples prepared by the traditional extraction method. Application of ultrasonication-assisted extraction procedure as follows: 12.1, 17.9, 10.5, 9.6 and 14.6, 19.7, 11.6, and 10.7 mg/g of extract, respectively. The quantity of 6-gingerol was as follows: 10.2, 15.1, 7.3, 6.9 and 12.7, 17.8, 8.8, and 7.9 mg/g of extract, respectively.	(Foudah et al., 2020)

GC & GC/MS	Mature ginger (Z. *officinale* Roscoe) rhizomes	Investigate the effect of steam distillation versus liquid CO_2_ samples on the formation of the volatile aldehydes and ketones from non-volatile gingerols	The non-volatile gingerol thermal degradation resulting from steam distillation was avoided by extraction at low temperature using liquid carbon dioxide extraction.	(Chen, Rosen, & Ho, 1986)
	Dried and fresh ginger	Identification of volatile components in essential oils via GC–MS & chemometrics	Qualitative & quantitative detection of chemical in foods, herbal medicines, and environmental samples. In the dried sample, 140 compounds (62.8 %) were identified versus 136 (47.1 %) in fresh sample.	(Gong, Fung, & Liang, 2004)
	Ginger obtained from 7 major production areas in China	Distinguish varieties & geographical origins of dried ginger	Using Headspace gas chromatography-mass spectrometry (HS-GC–MS) & fast gas chromatography electronic nose (fast GC e-nose) aided to discriminate samples obtained from seven major production areas in China.	(Ahad et al., 2021, Yu et al., 2022)
	Fresh ginger rhizome	To identify best operating conditions for the production of spray-dried ginger oleoresin powder using RSM.	The best conditions for spray drying of ginger oleoresin powder were found as wall material (gum acacia & whey protein isolate) concentration (30 %), gingerol level (25 % for gum acacia and 23 % for whey protein isolate), inlet temperature (170 °C) with feed flow rate (350 mL/h).	(Ahad et al., 2021)

HPLC	Different ginger-containing supplements, spices, mints, beverage and teas	Establish a simple procedure for the assay of 6-, 8- & 10-gingerol	The HPLC method was accurate, reproducible, robust, and with no degradations of gingerols as in GC/MS. The recoveries of 6-, 8-, and 10-gingerol, and 6-shogaol were at 94.7, 93.6, 94.9, 97.1 %, respectively. The within-day coefficients of variation for 6-gingerol, 6-shogaol, 8-gingerol, and 10-gingerol standards were at 2.54, 2.38, 2.55, and 2.31 %, respectively.	(Schwertner and Rios, 2007)
	Different ginger samples (dried, baked & fresh)	Develop an accurate assay of gingerols	This technique detected trace levels of gingerols. LOD and LOQ were measured at 0.7525 and 3.010 µg/ml for 6-gingerol, 0.8841 and 2.947 µg/ml for 8-gingerol, and 0.9048 and 3.016 µg/ml for 10-gingerol, respectively.	(Li et al., 2008)
	Polyherbal formulation contain *Withania somnifera* & *Zingiber officinalis* extracts.	Develop a reversed-phase HPLC technique for the determination of 6-gingerol	Validation proved that the technique used is specific, accurate, precise, reliable, and reproducible. LOD and LOQ were at 0.2 μg and 1.0 μg for 6-gingerol, respectively.	(Jain et al., 2010)
	Several varieties of Indonesian ginger	Determination of 6-,8- and 10- gingerol and 6-shogaol	Good resolution, LOD and LOQ ranged from 0.034 − 0.039 mg/mL and 0.112–0.129 mg/mL, respectively.	(Rafi et al., 2013)
	Fresh rhizome part of 9 different ginger varieties	Quantitative analysis and separation of 6-gingerol	Dynamic range at 25–400 µg/mL; LOD 0.41 µg/mL and LOQ 1.25 µg/mL	(Suparna et al., 2023)
	11 Ginger containing commercial products	A sensitive RP- HPLC ECD procedure was employed for the assay of 6-, 8-, & 10- gingerols and 6-, 8-, & 10- shagoals.	This method provided remarkable sensitivity for the redox sensitive compounds as low as 7.3 –20.2 pg of LOD and a range of 14.5 to 40.4 pg of LOQ. ECD provides an on-line production of qualitative data and capacity to determine peaks due to different voltammetric characteristics.	(Shao, Lv, Parks, Wu, Ho, & Sang, 2010)
			
	21 Extracts from different ginger type (fresh versus dried) using different solvents, extraction methods.	Determination of gingerols & 6-shagoal levels.	A chromatographic fingerprinting HPLC-DAD validated method revealed differences among ginger extracts	(Liu et al., 2014)
	Gingers from 5 diverse ginger-producing countries e.g. China, India, Malaysia, Thailand and Vietnam	Discrimination of ginger samples	HPLC fingerprinting resolved the overlapping gingerol peaks of a complex system without the need for optimization aided by chemometric tools. A successful mathematical model with high prediction power, with chemical markers for each source identified.	(Yudthavorasit, Wongravee, & Leepipatpiboon, 2014)
	Ginger samples, applying different extraction conditions *viz.* different solvents, methods and extracting time	Assay of gingerols and 6-shagoal	A reliable and rapid HPLC coupled with PDA was established and validated. LODs and LOQs of gingerols ranged from 0.024 − 0.366 μg/mL and 0.073–––1.110 μg/mL, respectively while the mean recoveries ranged between 95.50 % and 104.14 % with RSDs less than 1.27 %.	(Wang et al., 2018)
	10 Samples in all forms of either dietary supplements (tablet, capsule, soft gel) or dietary ingredients (rhizome powder, dry extract, and oleoresin).	Quantification of non-volatile	Validation confirmed linear response (R2 > 0.999), accuracy (91.1–103.2 %, spike recovery), precision (RSD < 5 %), and robustness.	(You et al., 2019)
	Determination of gingerols in a variety of dietary supplements of different dosage forms	Quantification of 6-,8- and 10-gingerols and 6-, 8- and 10-shagoals	An analytical method was validated using HPLC coupled to UV–Vis. The procedure proved to be selective, linear (R2 > 0.999), specific, accurate (91.1–103.2 % spike recovery rate), and precise (RSDr < 5 %, RSDR < 8 thus meeting required standards.	(You et al., 2020)
	Rhizome of *Z. officinale*	Assay of 6-gingerol & 6-shogaol levels	UPLC presented an additional value for gingerols profiling aided by improving extraction process using RSM.	(Ghasemzadeh, Jaafar, & Rahmat, 2015)
	98 Samples of dried Australian processing-grade ginger	Assay of 6-gingerol, 8-gingerol, 10-gingerol and 6-shogaol	The 6-gingerol content was correlated with 8-gingerol, but only weakly correlated with 6-shogaol. The level of variation in 6-shogaol was less than that observed among gingerols.	(Johnson et al., 2021)

**LC/MS**	Fresh commercial ginger	Assay of gingerols using HPLC–UV & HPLC-ESI-MS–MS	6-Shogaol was detected in fresh ginger at low level, versus higher levels of 6-, 8- and 10-shogaol in ginger oleoresin due to their transformation from gingerols.	(He, Bernart, Lian, & Lin, 1998)
	Ginger raw herb & samples of dried aqueous extract	Validate a method for the assay of gingerols	This method was validated to detect 6-, 8-, 10-gingerol, and 6-shogaol in ginger and its dried aqueous extract. Average recovery was at 97 % with a RSD < 8 %. L.O.D. for 6-, 8-, 10-gingerol, and 6-shogaol in the raw herb were at 0.22, 0.04, 0.09, and 0.07 mg/g, respectively, while in the dried aqueous extract at 0.11, 0.02, 0.02, and 0.14 mg/g, respectively	(Lee et al., 2007)
	Dried ginger powder, fresh ginger powder, ginger tea products and hot water ginger extracts	Simultaneous separation, identification, & quantification of gingerols	LC-TOF/MS/MS was established for the fingerprint of ginger nutritional supplements	(Tao, Li, Liang, & Van Breemen, 2009)
	Ginger products (fresh, powdered tea products, hot water ginger extracts)	Assay of gingerols	The method showed adequate linearity (r > 0.999) in a wide range (5–5000 ng/mL), low LOD and LOQ, high precision, and inter- and intraday repeatability. The sensitivity level for gingerols and shogaols detection by TOF/MS was much higher than UV.	(Park & Jung, 2012)
	Fresh, powdered dry gingers, and ginger dietary supplements	Assay of gingerols	UPLC-ESI-MS/MS provided fast detection of gingerols in positive ion mode with ammonium formate as modifier showing higher (4.5- to 15.7-fold) sensitivity level than negative ion UPLC. Positive ion UPLC-MS/MS displayed outstanding linearity (r2 > 0.999), low LOD = 2.5 to 8.2 pg, high accuracy and precision, and with insignificant matrix effect.	(Farag et al., 2022, Park and Jung, 2016)
	12 *Z. officinale* varieties from India	Assay of gingerols	LOD and LOQ for 6-gingerol, 8-gingerol, and 10-gingerol were at 0.92, 0.85, & 1.07 ng/mL, & 2.95, 2.72, & 3.01 ng/mL, respectively.Patna and Lucknow varieties comprised the highest level of gingerols.	(Ashraf, Mujeeb, Ahmad, Ahmad, Ahmad, & Amir, 2014)
	Oral administration of ginger extract within plasma and tissues of rats	Determination of the pharmacokinetics & tissue distribution of 6-, 8- & 10-gingerols & 6-, 8-, & 10-shagoals	A validated UPLC-Q-exactive–HRMS) method separated analytes with (LLOQ) was 1.0 ng/mL. e *T*-max of gingerols/shogaols indicated that shogaols were absorbed faster in relative to the carbon chain length. The t _1/2_ of shogaols were longer than gingerols.At 0.33 h post oral administration, 6-gingerol, 6-shogaol, 8-gingerol 8-shogaol and 10-gingerol were detected in rat tissues. The method was found linear for all gingerols with R^2^ above 0.9910.	(Li et al., 2019)
	Fresh ginger samples or dried and commercial products (rhizomes, extracts, commercial tea samples, based ginger candies, thermogenic supplements, and tonic water from Brazil)	Develop a practical, fast, efficient, high throughput, & eco-friendly sample preparation method for the assay of gingerols	Graphene oxide/magnetite (GO-Fe_3_O_4_) nanocomposite was used as a sorbent for magnetic solid phase extraction (MSPE) of 6-, 8-, and 10-gingerols. The whole GO-Fe_3_O_4_-MSPE-LC-MS/MS technique showed high selectivity with LOD ranging from 2 − 3 μg/L. Intra-day and inter-day RSDs ranged from 1.7 − 13.4 % and 0.4–10.9 %, respectively.	(Akamine et al., 2021)

Electrochemical detection	Commercially available crushed ginger	Detection of gingerol compounds using adsorptive stripping voltammetry (AdsSV)	Electrochemical detection offered fast detection method for QC purposes using a multi-walled carbon nanotube modified basal plane pyrolytic graphite electrode. Detection level at a linearity range from 1 µM to 50 µM, with LOD of 0.21 µM and LOQ of 0.71 µM, respectively.	(Chaisiwamongkhol, Ngamchuea, Batchelor-McAuley, & Compton, 2016).
	Commercially available ginger	Electroanalytical quantification of gingerols using cyclic voltammetry with polished glassy carbon electrodes.	Identification and characterization of gingerols and shogaols	(Idris, Yasin, & Usman, 2019)
	Nonfumigated ginger and sulfur-fumigated ginger samples	Identification and quantification of gingerols and 6-shogaol using FT-NIR	FT-NIR offered a consistent procedure to evaluate ginger batches qualitatively.FT-NIR acts as a substitute to HPLC in the detection of gingerols. PLS and CP-ANN were employed to detect gingerols in S- and NS-ginger.	(Yan et al., 2021).

### 3.1 High performance thin layer chromatography (HPTLC)

High performance thin layer chromatography (HPTLC) is a rapid and robust method commonly used in laboratories for drugs screening. A sensitive and accurate technique has been elaborated to detect 6-gingerol from three diverse sources of ginger using mobile phase*, n*-hexane, and diethyl ether (40: 60 v/v) thus allowing estimation of 6-gingerol with good resolution and separation from additional components and is relatively simple suiting the large screening purposes (Rai et al., 2006). Further, Melianita et al. (2007) established a fast, simple densitometric procedure for the detection of 6-, 8-, 10-gingerols and 6-shogaol, which can be used for systematically in industrial quality control laboratories. Different varieties of ginger were extracted with methanol and further chromatographed on precoated HPTLC plates eluted with toluene–ethyl acetate (3:1, v/v) as mobile phase. Quantitative assessment was done by determining the absorbance reflectance of the analyte at λ_577_ nm after spraying with anisaldehyde-H_2_SO_4_ reagent. HPTLC advantages include its robustness allowing for the analysis of several samples at the same time using minimal amount of mobile phase different from HPLC thus reducing duration and cost of analysis (Melianita, Witha, Arifin, Kartinasari, & Indrayanto, 2009).

The ultrasonic assisted fresh rhizome samples were chromatographed on RPTLC pre-coated with RP-18 60F_254_ as the stationary phase developed using acetonitrile–water–formic acid (7:2:1 v/v/v) and scanned at 500 nm. Separation of all 3 gingerol bands was achieved from spots for gingerols (R_f_ of 6-gingerol 0.73, 8-gingerol 0.59 and 10- gingerol 0.36) (Khan et al., 2010). HPTLC technique was developed for the quantification of 8-gingerol in an assortment of ginger-containing products with good linearity at a concentration range of 50–500 ng/spot, accuracy of 98.2 % with RSD 0.70–1.41 posing it as validated method for 8-gingerol quantification (Alam, 2013).

For the concomitant detection of 6-shagaol and 6-gingerol within the conventional and ultrasonic-assisted extracts of ginger rhizome and its commercial products viz, ginger powder, capsules, and ginger teas, a fast, simple, and low-cost green RP-HPTLC densitometry technique was developed on RP-18 HPTLC plates using ethanol: water (6.5:3.5 v/v) as a mobile phase. The study was accomplished at λ max 200 nm and confirmed by detection of band at R_f_ = 0.36 ± 0.01 for 6-shagaol, 0.53 ± 0.01 for 6-gingerol in comparison to their standards. The studied technique was proven to be precise, linear, accurate, robust, and sensitive for the concurrent quantification of 6-shagaol and 6-gingerol. The quantity of 6-shagaol in different samples as the conventional extracts of ginger rhizome, and its commercial products as ginger powder, capsules, and ginger teas was found to be at 12.1, 17.9, 10.5, and 9.6 mg/g of extract, respectively while samples prepared by ultrasonication-assisted extraction method were estimated at 14.6, 19.7, 11.6, and 10.7 mg/g of extract. Meanwhile, the quantity of 6-gingerol within the aforementioned samples was detected at 10.2, 15.1, 7.3, 6.9, 12.7, 17.8, 8.8, and 7.9 mg/g of extract, respectively (Foudah et al., 2020).

### 3.2 Gas chromatography (GC) & gas chromatography coupled to mass spectrometry (GC/MS)

Whilst considered sensitive for the detection of gingerols and shogaols, there are several limitations associated with GC and GC/MS techniques for analyzing gingerols considering their thermal instability. Gas operating column temperatures led to a significant transformation of 6-gingerol into 6-shogaol as mentioned using 3 % SE-30 column. Another attempt was reported using carbowax column to determine gingerols level in either steam distilled versus liquid carbon dioxide samples, thermal degradation occurred during preparation by steam distillation thus altering its level in comparison with liquid carbon dioxide thus affecting ginger ultimate flavor (Chen et al., 1986, Chu-Chin and Chi-Tand, 1987).

A method of analysis was established for detecting volatiles in essential oils extracted from both fresh and dried ginger via GC–MS analyzed using chemometric tools. Chemometrics provided improved resolution of the two-dimensional dataset from GC–MS where the drifting baseline could be amended. Chemometric resolution according to the two-dimensional data enhanced the separation capacity of GC/MS and aided in the qualitative and quantitative detection of closely related isomers as in gingerols, and is suited for analysis in complex matrices *viz.* foods, herbal medicines, and environmental samples (Gong et al., 2004). This study managed to enhance the operative conditions for the advancement of spray-dried ginger oleoresin powder by using response surface methodology (RSM) to assess the impact of procedure settings on the quality specifications of ginger oleoresin powder. The responses altogether were considerably influenced using the tested variables. The isolated whey protein proved to be the greatest influential factor on the production of ginger oleoresin powder with the highest process yield (22.51–73.19 %), less hygroscopic (21.73 %), high rehydration ratio (48.61–88.75 %), and oleoresin content (6.57–9.93 %) compared to gum acacia. RSM identified ideal settings for spray drying of ginger oleoresin extract with preferable characteristics and suggested that the acquired example can be implemented for the mass manufacture of oleoresin powder for both food and nutraceutical industries (Ahad et al., 2021).

### 3.3 High performance liquid chromatography (HPLC)

Different attempts were made for detecting gingerols and shogaols using HPLC with validation of most methods to ensure accuracy, precision, and reproducibility. For example, HPLC was used to analyze 6-, 8-, and 10-gingerols in ethyl acetate samples extracts of nutraceuticals containing ginger, mints, teas, spices, and beverage extracted and analyzed using C-8 reversed phase column eluted using mobile phase methanol: water (65:35, v/v) and UV detector at 282 nm. The recoveries of 6-, 8-, and 10-gingerols, and 6-shogaol were at 94.7, 93.6, 94.9, 97.1 % from ginger dietary supplements, respectively. The within-day coefficients of variation for 6-gingerol, 6-shogaol, 8-gingerol, and 10-gingerol standards were at 2.54, 2.38, 2.55, and 2.31 %, respectively (Schwertner & Rios, 2007). Simultaneous determination of gingerols in different ginger samples (dried, baked & fresh) through HPLC-DAD using a gradient elution of acetonitrile–water on C18 column. LOD and LOQ were measured at 0.7525 and 3.010 µg/mL for 6-gingerol, 0.8841 and 2.947 µg/mL for 8-gingerol, and 0.9048 and 3.016 µg/mL for 10-gingerol, respectively. This procedure can detect trace amounts of gingerols in biofluids (Li et al., 2008). In another study, a reversed-phase HPLC technique was implemented for the detection of 6-gingerol within herbal formulation including extracts of *Withania somnifera* and *Z. officinalis*. HPLC analysis was carried out using C18 column and acetonitrile and water 40: 60 (v/v) as eluent coupled to UV detection. In agreement with (ICH) International Conference on Harmonization guidelines, validation results showed that the specificity, accuracy, precision, reliability, and reproducibility of the method. LOD and LOQ were at 0.2 µg/mL and 1.0 µg/mL for 6-gingerol, respectively (Jain et al., 2010).

Using reversed phase capillary LC for the concurrent determination of 6-,8- and 10- gingerol and 6-shogaol in several varieties of Indonesian ginger was developed on C30 (stationary phase) using 60 % acetonitrile (mobile phase). These resulted in the separation of the 4 analytes within 25 min showing good resolution and LOD and LOQ ranged from 0.034 − 0.039 mg/mL and 0.112–0.129 mg/mL, respectively. The concurrent quantitative analysis in addition to chemometric method *viz.* discriminant analysis was demonstrated to be an effective tool for the quality control analysis of raw ginger and ginger products (Rafi et al., 2013). Quantitative analysis of 6-gingerol was performed using a C18 RP column (250×4.6 mm, 5 μm) and it was eluted using mobile phase consisting of solvent A (methanol) and solvent B (1 % acetic acid) at a flow rate of 1.0 mL/min. The method was validated for 6-gingerol with a dynamic range of 25–400 µg/mL, LOD 0.41 µg/mL and LOQ 1.25 µg/mL (Suparna et al., 2023).

Another reversed-phase HPLC electrochemical based technique was developed for the determination of gingerols and shogaols in 11 commercial ginger products. HPLC integrated with an electrochemical array detector (ECD) provided high sensitivity for redox sensitive compounds in comparison to UV and MS detectors, and suiting it for the detection in biofluids at trace levels. Asides, ECD also offered an on-line production of qualitative data and the capability to sort out peaks due to various voltametric attributes. The very high sensitivity was achieved as low as 7.3 –20.2 pg of LOD and 14.5–40.4 pg of LOQ, posing it for the detection of gingerols in biofluids and pharmacokinetic studies in the future. Another benefit of ECD in comparison to LC/MS lies in the clean cell activity using high electro potential in ECD cells following each run, indicating the potential of using it for the analysis of the biofluids and tissue samples. Clean cell activity is utilized for applying a high electro potential to the cells briefly, thus preventing samples against contamination and cleaning electrode surfaces (Jones & Zweier, 2014).

A chromatographic fingerprinting HPLC-DAD validated method was established to determine gingerols and 6-shogaols in ginger extracts. 21 Extracts were prepared from ginger (fresh versus dried) using different solvents, and extraction techniques. ANOVA Analysis manifested the effects of extraction methods on gingerols’ yield in that ascending order: high pressure with high temperature, blender, and low pressure. Best solvent used to isolate gingerols was 95 % ethanol, while ginger type exerted also significant effects on gingerols level, but less dramatic compared to solvent type. With the intention of increasing gingerols’ extraction yield, a blend of dry ginger, 95 % ethanol, and high pressure-high temperature extraction procedure ought to be employed, especially at an industrial level (Liu et al., 2014). HPLC fingerprint analysis was carried out on C18 column (3.9 × 150 mm, 5 µm) for profiling of ginger samples obtained from 5 different regions e.g., Malaysia, China, Thailand, Vietnam, and India, to detect source of ginger mostly based on gingerols profile. As a result, the differences in HPLC profiles yielded distinguishable pattern of each origin as visualized using similarity analysis, HCA, PCA and linear discriminant analysis (LDA). The profiles of ginger were also clustered and separated depending on the geographical proximity of the countries of origin. Furthermore, a successful mathematical model with high prediction power was developed and chemical makers were recognized for each origin (Yudthavorasit et al., 2014).

A reliable and rapid HPLC method coupled with PDA was established and validated to determine gingerols and 6-shogaol levels from ginger samples, applying different extraction methods *viz.* different solvents, extraction methods and time in order to optimize for extraction conditions. Best results were achieved using methanol and ultrasonic for 20 min. According to Anisa et al. (2014), soxhlet extraction using ethanol provided the maximum yield of 6-gingerol (8.40 mg/g) at 78.1 °C for 8 h with a solvent to solid ratio of 4:1 (Anisa, Azian, Sharizan, & Iwai, 2014). In a further study, Said et al. (2015) compared different extraction methods *viz.* soxhlet and ultrasonic-aided extraction method for the isolation of 6-gingerol using a variety of solvents and different temperatures. Results indicated that maximum isolation yield of 6-gingerol (7.3 % w/w) was achieved using methanol, subsequently acetone, ethanol, and *n*-hexane at 64 °C (Said, Arya, Pradhan, Singh, & Rai, 2015). The LODs and LOQs of gingerols ranged from 0.024 − 0.366 and 0.073–1.110 μg/mL, respectively while mean recoveries ranged from 95.50 % and 104.14 % with RSD less than 1.27 % (Wang et al., 2018).

The compendial United States Pharmacopeia (USP-NF) monograph and International Organization for Standardization (ISO) 13685–1997 methods currently analyze gingerol at 282/280 nm. However, an HPLC-UV/Vis technique was developed to examine gingerols and shogaols at 230 nm demonstrating a higher sensitivity and greater peak resolution. The running time was effectively decreased by 2 min without the need of employing MS for analytes detection. It is the first method meeting all the requirements assigned by the AOAC (2017.012) and can be used as a reference procedure for FDA's cGMP agreement for the production and quality control of nutritional supplements (You et al., 2019). Another well-validated analytical procedure has been used for the estimation of 6-,8- and 10-gingerols and 6-, 8- and 10-shogaols using HPLC coupled to UV–Vis adopting AOAC guidelines for single-laboratory validation. The analysis was done using a C18 column (reverse-phase superficially porous particle), and gradient elution within a comparatively short time (12 min) and designed to identify gingerols within an assortment of nutritional supplements matrices *viz.* tablet, dry extract, capsule, powder, soft gel capsule, liquid capsule, and oleoresin. The method was found to be selective, linear (R^2^ > 0.999), specific, accurate (91.1–103.2 % spike recovery rate), precise (RSDr < 5 %, RSDR < 8 %) and thus fulfilled all AOAC Standard Method Performance Requirements (2017.012) criteria (You et al., 2020).

6-, 8-, 10-Gingerols and 6-shogaol were quantified in 98 samples of dried Australian processing-grade ginger using HPLC. Significant variation was found in gingerol and shogaol contents of ginger samples grown under different conditions. 6-Gingerol level was found correlated with 8- and 6-gingerol content, but only weakly correlated with 6-shogaol. The level of variation in 6-shogaol content was less than that observed in case of gingerols contents suggestive that zeroth-order reaction kinetics under mild dehydration conditions could be responsible for this observation (Johnson et al., 2021).

The ultra-performance power of (UPLC) presented an additional value for gingerols profiling compared to HPLC, further aided by improving extraction technique using response surface methodology RSM by comparing UV absorbance. Response surface methodology allowed the optimization of 6-gingerol and 6-shogaol assay within ginger extracts. The outcomes indicated that time needed for extraction and the extraction temperature significantly influenced 6-gingerol and 6-shogaol yields. Reflux extraction of ginger rhizome at 76.9 °C for 3.4 h was shown to be the utmost effective settings for 6-gingerol and 6-shogaolextraction (Ghasemzadeh et al., 2015).

A recent study optimized the conditions for the separation of 6-, 8-, 10-gingerols and 6-shogaol from a red ginger extract using HPLC along with optimization of composition of the mobile phase, flow rate and UV wavelength. Johan Sukweenadhi and Kartini, (2023) allowing for complete separation of all isomers.

### 3.4 Liquid chromatography-mass spectrometry (LC-MS)

Detection of gingerols from raw herb using sonication and analyzed using C18 column eluted with water-acetonitrile gradient mobile phase coupled to PDA detection was employed. This method was validated to detect 6-, 8-, 10-gingerol, and 6-shogaol in both dried aqueous extract of ginger and its root. Identification was accomplished using negative electrospray ionization based on tandem MS data. Average recovery was at 97 % with an RSD < 8 %. LOD for 6-, 8-, 10-gingerol, and 6-shogaol in the dried aqueous extract were at 0.11, 0.02, 0.02, and 0.14 mg/g, while in fresh drug were at 0.22, 0.04, 0.09, and 0.07 mg/g, respectively (Lee et al., 2007).

A high resolution (LC-TOF/MS/MS) was conducted for the concurrent separation, identification, and quantification of gingerols from ginger products. Identification of gingerols and shogaols in the negative ion MS mode was based on fragmentation of the C4-C5 bond producing a neutral loss of 194 amu as characteristic fragment to be used for MS/MS based identification of gingerols in unknown samples. Thus, developing an acceptable chemical MS based profiling that can be used for the analysis of ginger dietary supplements (Tao et al., 2009).Park and Jung further developed (LC-TOF/MS) method for the analysis of gingerol-related compounds within ginger products. The accepted procedure showed adequate linearity (r > 0.999) at a wide range (5–5000 ng/mL), low LOD and LOQ, high precision, and inter- and intraday repeatability. The detection sensitivity of gingerols and shogaols using TOF/MS was 70–100 times greater than traditional UV detection at 288 nm (J. S. Park & Jung, 2012). UPLC electrospray ionization (ESI)-tandem mass spectrometry (MS/MS) method presents a potential platform in natural products profiling especially for the identification of novel gingerols and their metabolites in biotransformation studies (Farag et al., 2022). UPLC-MS/MS provided both fast separation and quantification of gingerols (4-, 6-, 8-, 10-, and 12-gingerols) and 6-, 8- and 10- shogaols within ginger products. Ionization polarity and mobile phase modifier were found to affect sensitivity of analytes detection exemplified by 0.05 mM ammonium formate. Interestingly, positive ion UPLC-MS/MS with ammonium formate in mobile phase as modifier to show greater (4.5- to 15.7-fold) sensitivity than negative ion mode, although gingerols as phenolics are readily to be detected and ionized in negative ion mode. Further, positive ion UPLC-MS/MS technique exhibited outstanding linearity (r^2^ > 0.999), low LOD 2.5 to 8.2 pg, high accuracy and precision, and insignificant matrix effect posing it for pharmacokinetic based studies targeting gingerols. Baseline separation of the 8 targeted gingerols and shogaols was accomplished in 1 min using UPLC with a short C18 core-shell column (Park & Jung, 2016).

UHPLC separation of gingerols in 12 ginger varieties (India) was analyzed isocratically using C18 column and acetonitrile: water (90:10 v/v) as eluent. The linear dynamic range was achieved within a concentration range of 2 – 1000 ng/mL for all analytes, and LOD and LOQ for 6-, 8- and 10-gingerols were at 0.92, 0.85, and 1.07 ng/mL, and 2.95, 2.72, and 3.01 ng/mL, respectively. Significant variation (0.176 %–0.290 % *w/w*) in gingerols level among the 12 varieties was observed with Patna and Lucknow varieties to encompass the maximum level identified as superior source of gingerols (Ashraf et al., 2014). A validated (UPLC-Q-Exactive–HRMS) technique was effectively used to assess the pharmacokinetics and tissue distribution of 6-, 8- and 10-gingerols and 6-, 8-, and 10-shogaols within plasma and tissues of rats post oral administration of ginger extract. Using C18 column (100 x 2.1 mm, 1.7 μm) and gradient elution with acetonitrile and 0.1 % formic acid, the lowest LOQ was at 1.0 ng/mL. For MS condition, response in positive ion mode was higher than in negative mode and in agreement with results of (Park & Jung, 2016). The validated method was successfully utilized for the determination of all gingerols and shogaols in rat plasma and organ samples after taking 400 mg/kg ginger extract orally. The *T*-max of gingerols/shogaols demonstrated the fastest absorption of shogaols in accordance with the length of carbon chain which might account for their higher efficacy as previously noted. The t_1/2_ of shogaols were long lasting than gingerols showing that the former had much extended residence time inside the body which might exert potential biological activities than gingerols (Li et al., 2019).

A new, accurate, and reliable UPLC-MS/MS procedure combined with a single marker technique was developed and validated to determine the quality of the volatile oil of ginger herb obtained by three different extraction procedures. The proposed method showed good linearity, sensitivity, repeatability, accuracy, and sample stability for the determination of four gingerol components. The proposed method has the advantages of shorter analysis time and higher efficiency posing it to be used for future quality control of ginger, and presenting a reference method for the analysis of multi-index components in other nutraceuticals (Zhining Li et al., 2023).

Graphene oxide/magnetite (GO-Fe3O4) nanocomposite was used as a sorbent for the magnetic solid phase extraction (MSPE) of gingerols from fresh, dried or any commercial ginger products further determined using HPLC-MS/MS. The entire GO-Fe_3_O_4_-MSPE-LC-MS/MS technique showed high selectivity with LOD ranging from 2 and 3 μg/L. Intra-day and inter-day RSDs ranged amongst 1.7–13.4 % and 0.4–10.9 %, respectively (Akamine et al., 2021).

### 3.5 Infrared spectroscopy

Infrared (IR) technique was established to discriminate and quantify nonfumigated ginger and sulfur-fumigated ginger using Fourier transform near infrared spectroscopy (FT-NIR) combined with chemometrics to evaluate ginger batches of sulfur-fumigated ginger and nonfumigated ginger without special sample extraction. To develop quantitative approaches depending on partial least squares (PLS) and counter propagation artificial neural network (CP-ANN) from the FT-NIR, major gingerols were analyzed quantitatively using HPLC. Finally, PLS and counter propagation artificial neural network (CP-ANN (were used to quantify for gingerols in sulfur-fumigated and nonfumigated ginger. The FT-NIR results can present another technique alternative to HPLC for the estimation of active compounds in ginger. Advantages of IR compared to HPLC lies in that it can be directly applied on solid ginger samples without any extraction step and further without the time consumed in chromatographic separation of peaks. Near infrared spectroscopy (NIRS) is considered as an influential, rapid, simple and un destructive analytical technique with no usage of chemicals or complicated preparation of samples, directly applied on plant material with no any processing i.e., grinding or extraction prior to analysis. In addition, it is a simple operation, insignificant sample preparation, and fast measurement of a spectrum in few seconds. Chemometric methods coupled to spectroscopic techniques were used extensively to detect the inherent resemblances and alterations amongst foods by evaluating specific parameters (Yan et al., 2021) and has yet to be exploited for the QC of ginger products.

### 3.6 Electrochemical detection of gingerols and shogaols

The capability of electrochemical detection for the assessment of ginger drug was demonstrated to offer a facile and fast detection method for QC purposes using adsorptive stripping voltammetry (AdsSV) as a procedure used for gingerols’ identification. A multi-walled carbon nanotube modified basal plane pyrolytic graphite electrodes (MWCNT-BPPG electrode) was used at a linearity range from 1 µM to 50 µM with LOD of 0.21 µM and LOQ of 0.71 µM, respectively. The protocol for extraction can be accomplished *via* a simple procedure using ethanol thus saving time for sample preparation (Chaisiwamongkhol et al., 2016). In another study, electroanalytical quantification of gingerols using cyclic voltammetry with polished glassy carbon electrodes was reported (Idris et al., 2019).

AdsSV, Adsorptive stripping voltammetry; CP-ANN, Counter propagation artificial neural network; MSPE, magnetic solid phase extraction; MWCNT-BPPG electrode, multi-walled carbon nanotube modified basal plane pyrolytic graphite electrodes; PLS, partial least square; RSM, response surface methodology.

## 4 Gingerols delivery systems and formulations

**Fig 4:**
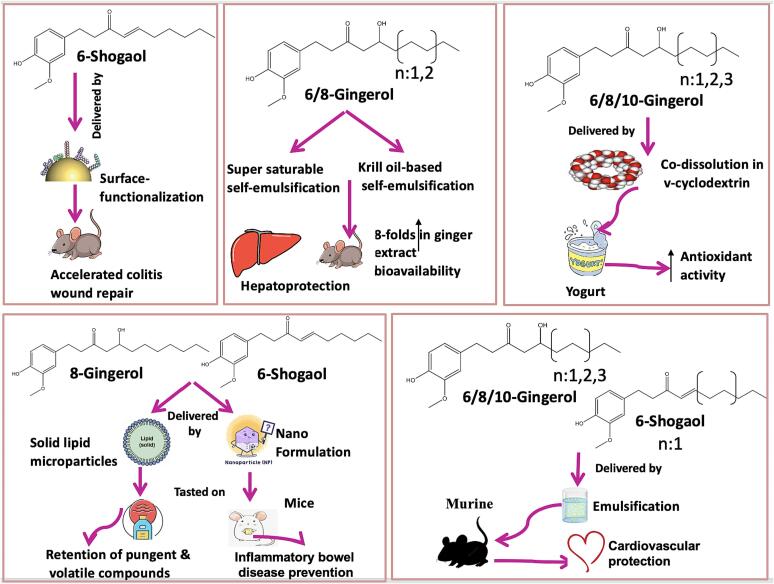
Representative examples of 6-shogaol surface-functionalization, (6-gingerol & 8-gingerol) super saturable self-emulsification and krill oil-based self-emulsification, (6-gingerol & 8-gingerol & 10-gingerol) co-dissolution in γ-cyclodextrin, (8-gingerol &6-shogaol) solid lipid microparticles and nano formulation, (6-gingerol & 8-gingerol & 10-gingerol & 6-shogaol) spontaneous emulsification*.*

Gingerols and shogaols are bioactive compounds that can readily degrade at certain environmental conditions and manufacturing steps during the ginger preparation. Accordingly, delivery systems are necessary to overcome these obstacles and ensure their stability (Suppl. Table. S1). Representative examples of gingerol’s delivery systems are shown in Fig. 4. A gingerol enriched extract to encompass 10-gingerol (26 %), 8-gingerol (20 %), and 6-gingerol (54 %) was formulated using γ-cyclodextrin prepared using co-dissolution method. A solid-state characterization of the γ-cyclodextrin gingerols showed that the inclusion compound features 1:1 host: guest stoichiometry as a powder in its microcrystalline form having crystalline cell of a tetragonal shape (Pais et al., 2017). Another study aimed at developing gingerol encapsulated in a niosomal nanoformulation to assess its potency as an antibacterial agent against carbapenem-resistant *Klebsiella pneumoniae* strains. The nanoformulation showed sustained release up to 70 h with high encapsulation efficiency. It was concluded that encapsulating gingerol in niosome augments its antibacterial/antibiofilm activities making it a potential strategy for drug delivery (Karbalaeiheidar & Ashrafi, 2023). Antioxidant assays including NO• scavenging, ABTS•+ scavenging, 5-LOX inhibition and β-carotene peroxidation identified γ-cyclodextrin as a suitable carrier for gingerols without affecting their reactivities. Yogurt as a food model system was assessed for the addition of gingerols/γ-cyclodextrin gingerols in food, with the color of fortified yogurt showing no change in organoleptic characters. Furthermore, yogurt encompassing γ-cyclodextrin gingerols exhibited an antioxidant effect consequently being appropriate for usage in nutraceuticals (Shukla et al., 2020a), Elastic nanovesicles were used to encapsulate 6-gingerol as a natural agent to decrease osteoporosis and musculoskeletal pain. The nanoformulation augmented skin absorption and drug release. It was concluded that gingerol gel formulation can treat pain ailments efficiently (Ghazwani et al., 2023). Furthermore, a micro-emulsifying carrier was formulated to improve the solubility and lower the surfactant: oleoresin ratio. The produced formulation consisted of 45.5 wt% of ginger oleoresin, that showed stability for a duration of 90 days under accelerated storage conditions. The formulation’s water dispersion showed high stability for a period of 24 h. In addition, an *in vitro* release study at a broad range of pH values revealed an augmentation by *ca*. 80 % in the solubility of ginger’s bioactive compounds versus pure form of ginger oleoresin. Lastly, addition of the prepared formulation in mango candy led to improvement in flavor and storage shelf-life at a temperature of 25 °C (Ogino et al., 2018, Shukla et al., 2020b). In addition, 6-gingerol loaded nano emulsification drug delivery was developed targeting better skin permeation for wound healing. The fabricated nanoformulation augmented both the solubility and skin penetration of 6-gingerol showing a better treatment of wound healing and anti-inflammatory properties compared to 6-gingerol being applied topically (Ahmad et al., 2023). Likewise, additional research aimed to improve ginger nutraceutical properties using super saturable self-emulsifying delivery techniques. The formulation improved ginger extract dissolution owing to micelle formation with a diameter of *ca*. 110 nm. Repeated oral administration of ginger extract/self-emulsifying drug delivery systems (100 mg ginger extract/kg) showed further hepatoprotective effects in mice models with carbon tetrachloride-induced hepatotoxicity. Self-emulsification drug delivery appeared efficacious for augmenting the nutraceutical properties of ginger extract (Ogino et al., 2022). In another study, optimization of gingerol-loaded niosomes for treating breast cancer was attempted. The drug/lipid ratios and the Span60/Tween60 proportions were modified resulting in a high release rate indicating the potential of this formulation of biocompatible niosomes encapsulating gingerol for treatment of breast cancer (Asghari Lalami et al., 2023). Other studies aimed to augment the nutraceutical characteristics of ginger extract using krill oil based self-emulsification drug delivery systems. Pharmacokinetic, physicochemical, and kidney protective characteristics of ginger extract formulations were examined. Glycerin promoted the production of a stable emulsion at acidic/neutral pH, producing 4-folds improved dispersion quantity of the active constituents in ginger. A polyurethane nanoparticle using spontaneous emulsification of ginger extract showed decreased irritation level on the skin of murine than the irritation level observed of pure ginger extract. The study further showed the decreased noxiousness of the ginger polyurethane nanoparticles and accordingly applicability of their usage as potential cardiovascular protective agent (Zhang et al., 2018). Gingerol-loaded nanoparticles were fabricated to assess its anti-cancer effects against gastric cancer cells. Release studies showed that there was a 55 % gingerol release after 21 h indicating a regular release of gingerol. Flowcytometry assay showed increase in apoptotic cells with gingerol nanoparticles suggestive that the anticancer effect of gingerol was mediated via induction of apoptosis (Kazazi et al., 2023). Other study employed a surface functionalizing method for the preparation of nanoparticles loaded with 6-shogaol. *In vivo*, oral administration of shogaol that was encapsulated into hydrogel systems (i.e., chitosan/alginate) considerably lessened colitis symptoms asides from the nanoformulation effect to accelerate colitis wound repair of mice model systems by controlling the expression levels of pro-inflammatory and anti-inflammatory factors. The study demonstrated that orally administered 6-shogaol delivery successfully accelerated colitis wound repair and represented an efficient therapeutic measure for treating inflammatory bowel diseases (Zhang et al., 2016). There is increasing demand for more effective treatments of chronic inflammatory bowel diseases (e.g., Crohn's disease and ulcerative colitis). In other studies, nanoparticles derived from ginger demonstrated efficient colon targeting after oral administration in mouse colitis model as manifested by improved intestinal repair, lessening of acute colitis, and prevention of chronic colitis/colitis-associated cancers. These nano formulations represented natural delivery for prevention/treatment of inflammatory bowel disease along with overcoming limitations such as limited production scale and potential toxicity (Puri et al., 2019). Additionally, ginger powder-loaded oil-in-water macroemulsion was prepared using olive-and silicone-oils. The objective was to assess the pain reduction extent of optimized ginger powder-loaded macroemulsion using an *in-vivo* dysmenorrhea mice. Only large doses of ginger powder-loaded macroemulsion that managed to restore uterine tissue's normal histomorphological structures (Oriani et al., 2016).

## 5 Conclusions and future directions

Gingerols and shogaols possess several biological activities which promoted their widespread usage in a diversity of industrial commodities. Several extraction methods were reported so far for gingerols’ recovery, such as conventional solvent extraction, subcritical water extraction, enzyme-assisted extraction, and ultrasound-assisted extraction. More modern extraction techniques were performed as well, among them, magnetic solvent extraction which has been receiving increasing attention as the magnetic sorbent is disseminated in the sample’s solution thus elevating the interfacial area among the sample and the sorbent and improving recovery yields. Although, different emerging technologies significantly improved CEO quality and quantity, future studies are required to address challenges facing the industrial application of these innovative technologies, and the exact mechanism by which gingerols being extracted need to be in-depth investigated. Although, most of the studies assessed the influence of a single variable on gingerols conversion to shogaols while the interactions between the variables have not been fully examined.

Comparative analysis of gingerols and shogaols regarding their health effects should be the next further step to aid identify best analogues targeting a certain effect. Several methods were attempted to detect gingerols and shogaols in different forms i.e., raw material whether fresh or dried, biofluids, pharmaceutical dosage forms *viz.* gels, tablets and capsules containing gingerols. Different chromatographic techniques, e.g., HPTLC, GC/MS, HPLC/UV, LC/MS, IR, and electrochemical methods have been used for the assay of gingerols and shogaols. HPTLC is the optimum method for the detection of gingerols as it is considered an accurate, rapid, simple, robust, and relatively cheap compared to hyphenated LC methods for gingerols assay in nutraceuticals. Moreover, it is considered a green/ecofriendly method due to the small amounts of organic/inorganic solvents used. Few attempts have been reported to quantify gingerols but sample preparation and exposure of ginger to heat may alter gingerolś level and the reason for not choosing the GC/MS as a method of choice for assay of gingerols due to the instability of gingerols which may change into shogaols thus altering the percentage composition of ginger whether fresh or dried. HPLC and UPLC are the mostly used techniques for analyzing gingerols and shogaols, with coupling of LC with MS to show improved detection especially suited for biofluids analysis. Several attempts using voltammetry and infrared for the assay of gingerols and shogaols have been reported in literature. Applying chemometric tools played an important role in discriminating different ginger samples from different sources and improving detection. Unfortunately, gingerols and shogaols can be easily degraded within severe environmental conditions and long production steps. Accordingly, delivery systems are necessary to ensure their stability, such as, co-dissolution, micro-emulsification, self-emulsification, spontaneous-emulsification, etc. Studies on gingerolś formulation are still needed considering their promising biological activities that can provide a wide array of health benefits. Identification of a reliable validated method for their identification is the best way to ensure their efficacy. Nevertheless, additional investigation is desirable, specifically in producing nutraceuticals and functional foods enriched with gingerols and shogaols at high bioavailability to provide the maximal health benefits. Assessment of the long-term administration of gingerols on humans should be the next logical steps aided by advances in omics that can identify any potential side effects or health risks based on changes in metabolome. With the advances in analytics using metabolomics technologies such as LC/SPE/MS/NMR ought to be considered to investigate metabolism and bioavailability of gingerols in human, more clinical studies on human volunteers are needed to be applied widely to identify metabolic activation or inactivation pathways of these bioactive in humans.

## Declaration of Competing Interest

The authors declare that they have no known competing financial interests or personal relationships that could have appeared to influence the work reported in this paper.

## Data Availability

No data was used for the research described in the article.
