# Supplementary material for: Gingerols and shogaols: A multi-faceted review of their extraction, formulation, and analysis in drugs and biofluids to maximize their nutraceutical and pharmaceutical applications
Source: Food Chem X. 2023 Oct 20;20:100947. doi: 10.1016/j.fochx.2023.100947 (PMC10739842; doi:10.1016/j.fochx.2023.100947)
Supplement: Supplementary data 1 [file mmc1.docx]

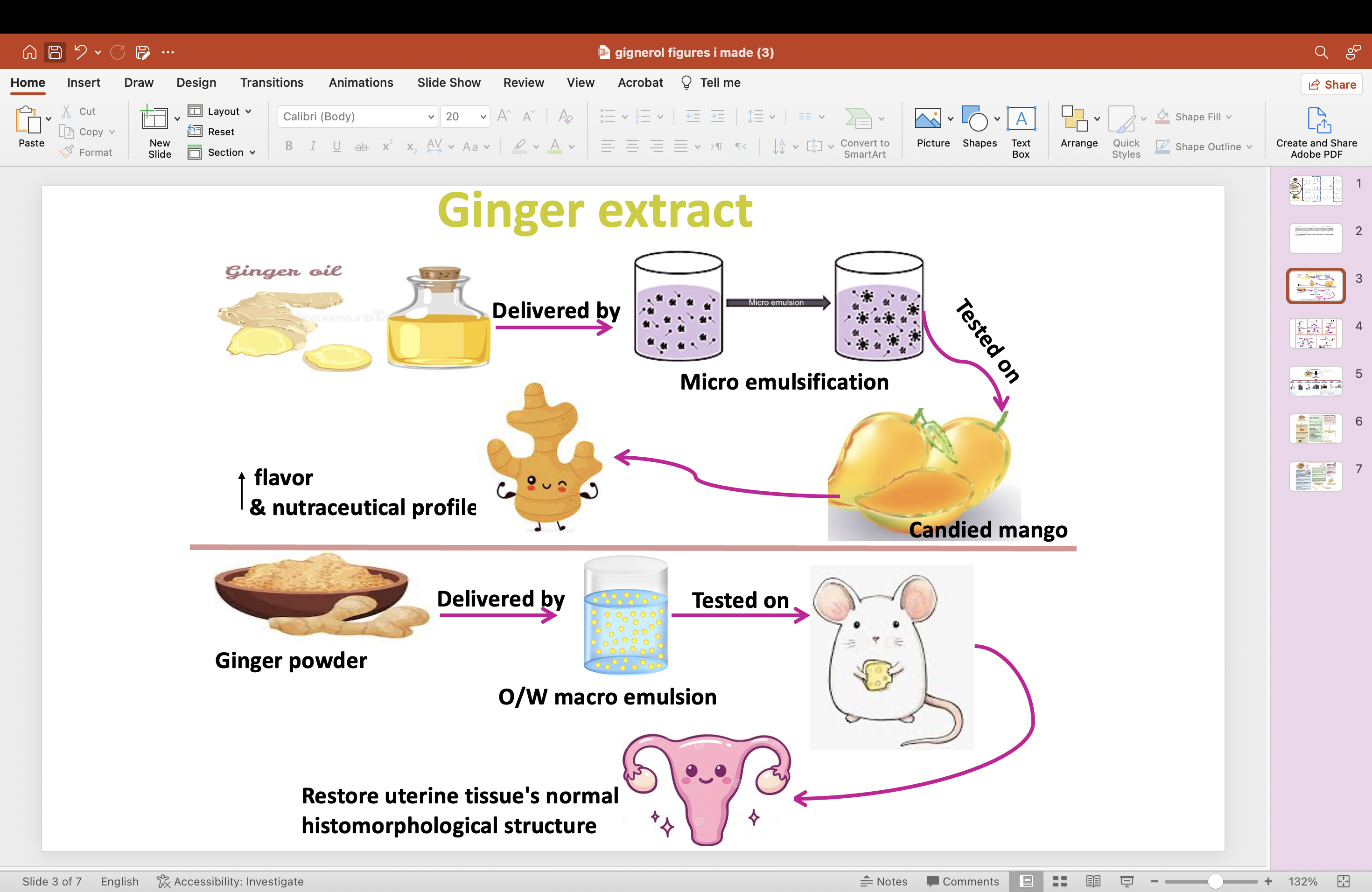


**Suppl. Fig. 1** Representative examples of ginger extracts micro emulsification & oil-in-water macro emulsions and their effects

**Suppl. Table S1.** Delivery systems of gingerols and ginger extracts, in food and health applications

| **Bioactive compounds** | **Delivery system** | **Test medium** | **Results** | **Reference** |
| --- | --- | --- | --- | --- |
| 6-Gingerol, 8-gingerol, & 10-gingerol | Co-dissolution in γ-cyclodextrin | Yogurt | Good antioxidant activity | (Shukla et al. 2020) |
| Ginger oleoresin | Micro emulsification | Candied mango | Enhancement of flavour & nutraceutical profile | (Ogino et al. 2018) |
| 6-Gingerol & 8-gingerol | Super saturated self-emulsification | Rats | Hepatoprotective effects | (Ogino et al. 2022) |
| 6-Gingerol & 8-gingerol | Krill oil-based self-emulsification | Rats | 8 Folds increase in ginger extract bioavailability | (Borcan et al. 2019) |
| 6-Gingerol, 6-shogaol, 8-gingerol & 10-gingerol | Spontaneous emulsification | Murine | Cardiovascular protection | (Zhang et al. 2018) |
| 6-Shogaol | Surface-functionalization | Mice | Accelerated colitis wound repair | (Zhang et al. 2016) |
| 6-Gingerol & 6-shogaol | Nanoformulation | Mice | Inflammatory bowel disease prevention | (Puri et al. 2019) |
| Ginger powder | Oil-in-water macroemulsions | Mice | Restore uterine tissue's normal histomorphological structure | (Oriani et al. 2016) |
